# Supplementary material for: A Humanized Yeast Phenomic Model of Deoxycytidine Kinase to Predict Genetic Buffering of Nucleoside Analog Cytotoxicity
Source: Genes (Basel). 2019 Sep 30;10(10):770. doi: 10.3390/genes10100770 (PMC6826991; doi:10.3390/genes10100770)
Supplement: Supplementary file 1 [file genes-10-00770-s001.zip › Additional_Files/Additional_File7_GOTermHeatmaps.zip!/Additional_File7_GOTermHeatmaps/DNA_packaging.pdf]

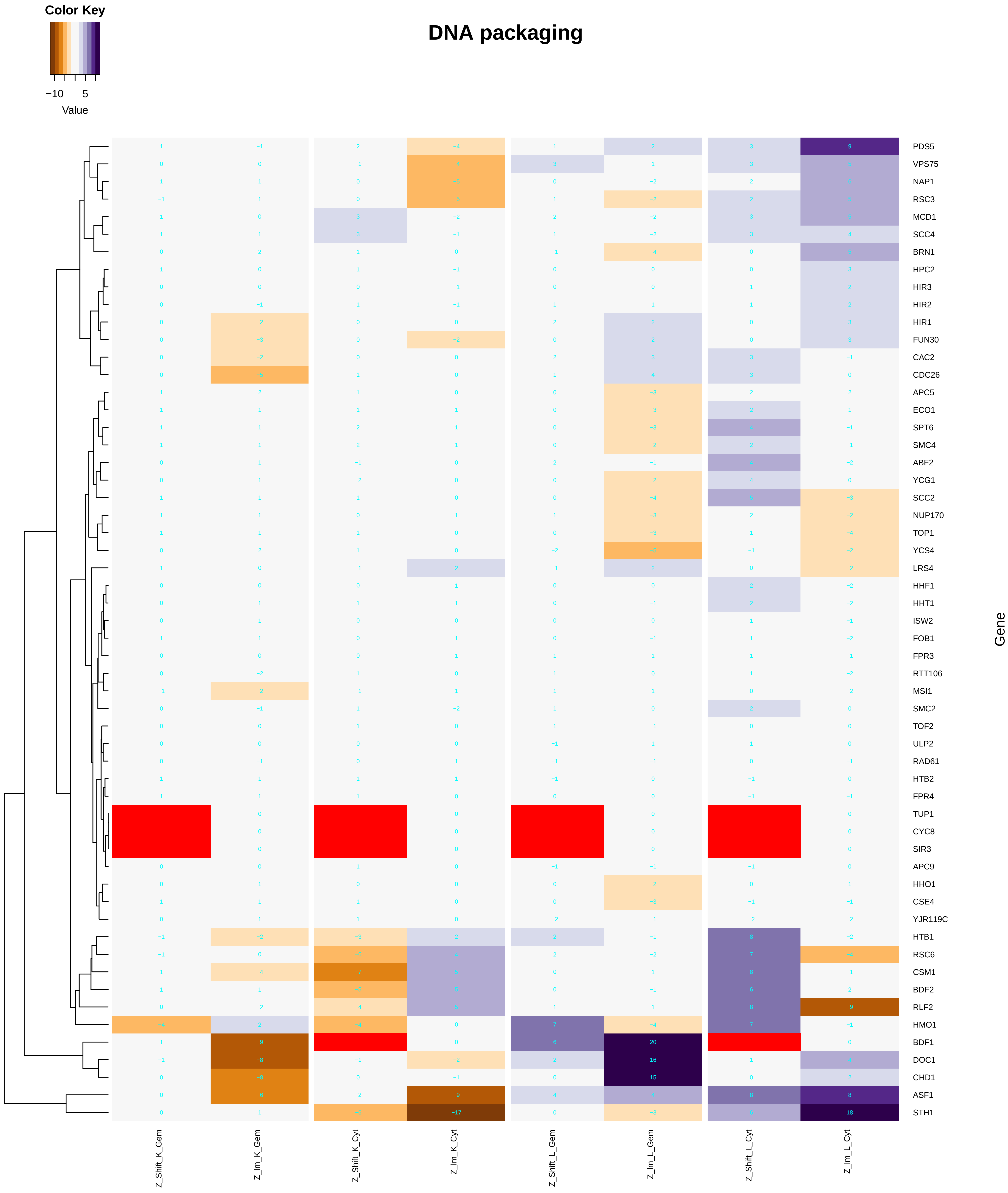
DNA packaging
Color Key
−10
5
Value
PDS5
VPS75
NAP1
RSC3
MCD1
SCC4
BRN1
HPC2
HIR3
HIR2
HIR1
FUN30
CAC2
CDC26
APC5
ECO1
SPT6
SMC4
ABF2
YCG1
SCC2
NUP170
TOP1
YCS4
LRS4
HHF1
HHT1
ISW2
FOB1
FPR3
RTT106
MSI1
SMC2
TOF2
ULP2
RAD61
HTB2
FPR4
TUP1
CYC8
SIR3
APC9
HHO1
CSE4
YJR119C
HTB1
RSC6
CSM1
BDF2
RLF2
HMO1
BDF1
DOC1
CHD1
ASF1
STH1
Gene
Z_Shift_K_Gem
Z_Im_K_Gem
Z_Shift_K_Cyt
Z_Im_K_Cyt
Z_Shift_L_Gem
Z_Im_L_Gem
Z_Shift_L_Cyt
Z_Im_L_Cyt

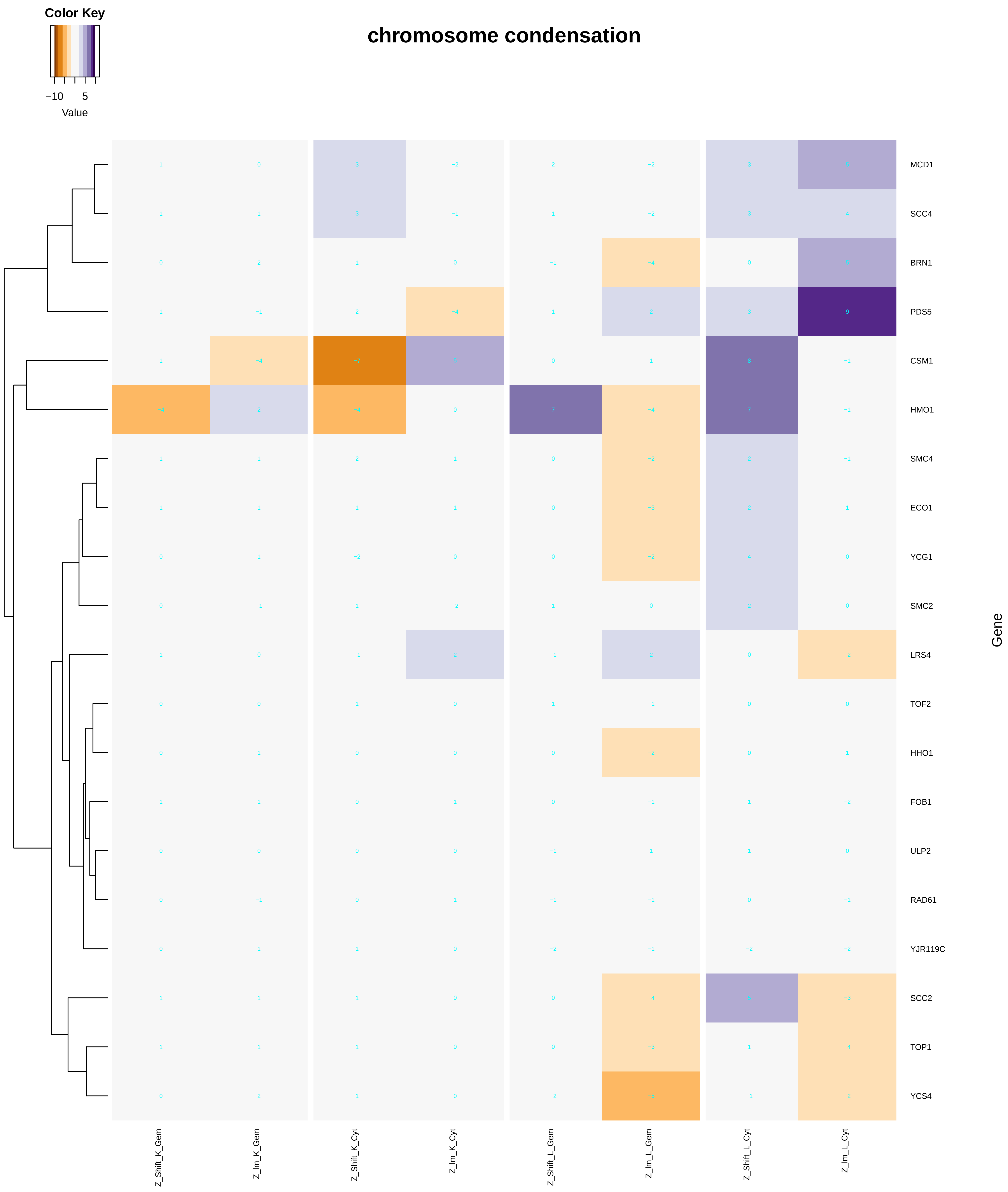
Color Key
−10
5
Value
chromosome condensation
MCD1
SCC4
BRN1
PDS5
CSM1
HMO1
SMC4
ECO1
YCG1
SMC2
LRS4
TOF2
HHO1
FOB1
ULP2
RAD61
YJR119C
SCC2
TOP1
YCS4
Gene
Z_Shift_K_Gem
Z_Im_K_Gem
Z_Shift_K_Cyt
Z_Im_K_Cyt
Z_Shift_L_Gem
Z_Im_L_Gem
Z_Shift_L_Cyt
Z_Im_L_Cyt

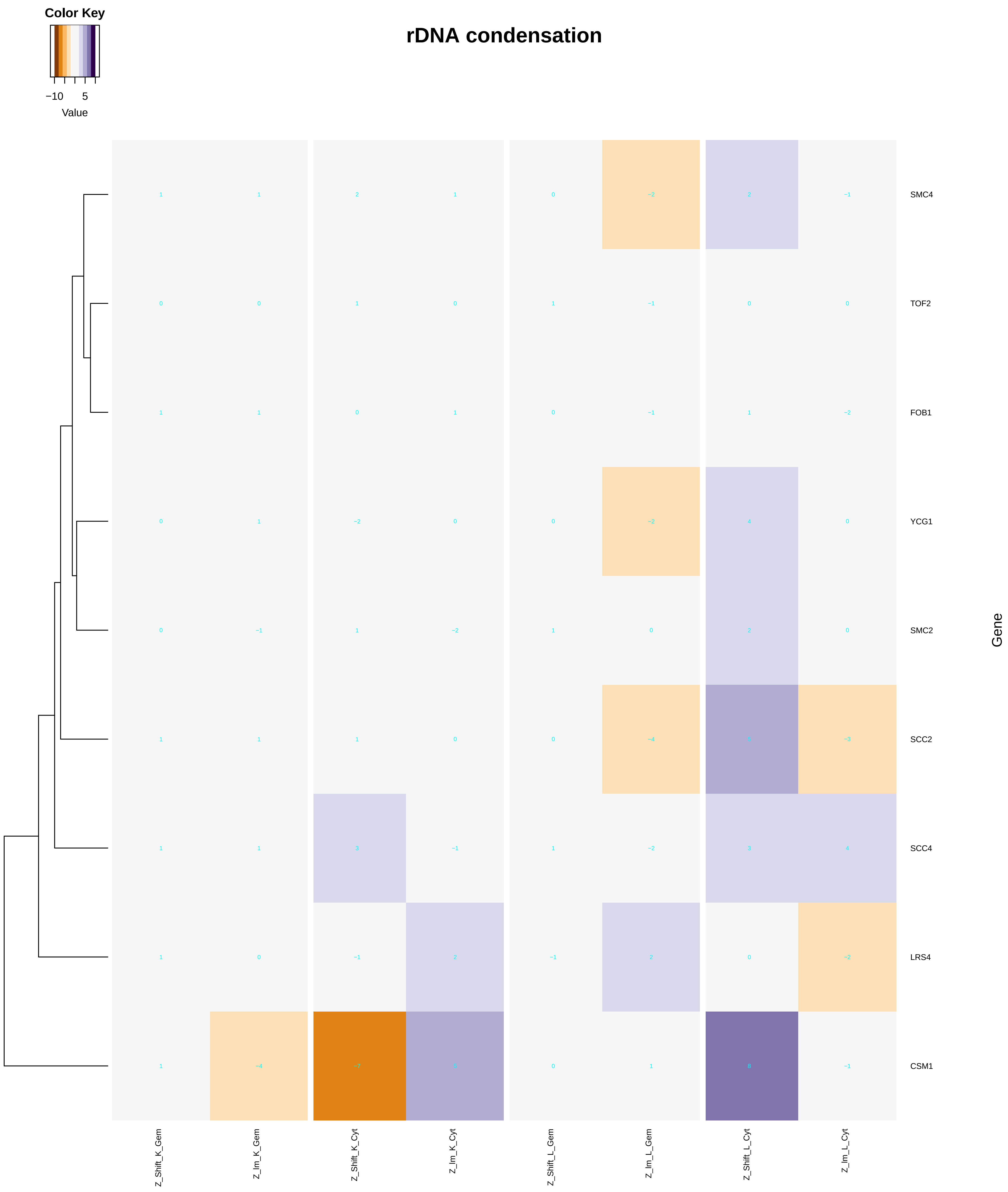

rDNA condensation
Color Key
−10
5
Value
SMC4
TOF2
FOB1
YCG1
SMC2
SCC2
SCC4
LRS4
CSM1
Gene
Z_Shift_K_Gem
Z_Im_K_Gem
Z_Shift_K_Cyt
Z_Im_K_Cyt
Z_Shift_L_Gem
Z_Im_L_Gem
Z_Shift_L_Cyt
Z_Im_L_Cyt

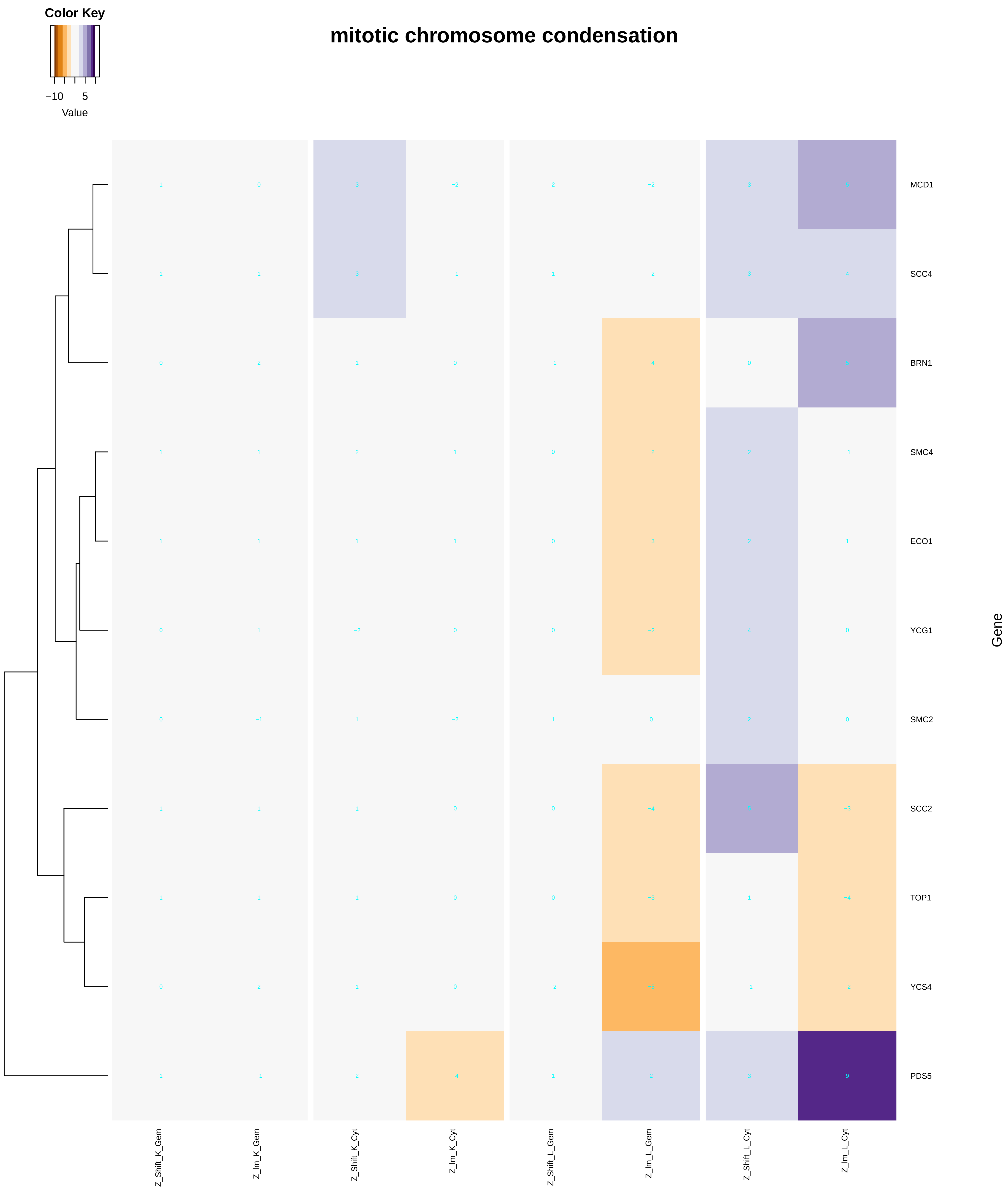
Color Key
−10
5
Value
mitotic chromosome condensation
MCD1
SCC4
BRN1
SMC4
ECO1
YCG1
SMC2
SCC2
TOP1
YCS4
PDS5
Gene
Z_Shift_K_Gem
Z_Im_K_Gem
Z_Shift_K_Cyt
Z_Im_K_Cyt
Z_Shift_L_Gem
Z_Im_L_Gem
Z_Shift_L_Cyt
Z_Im_L_Cyt

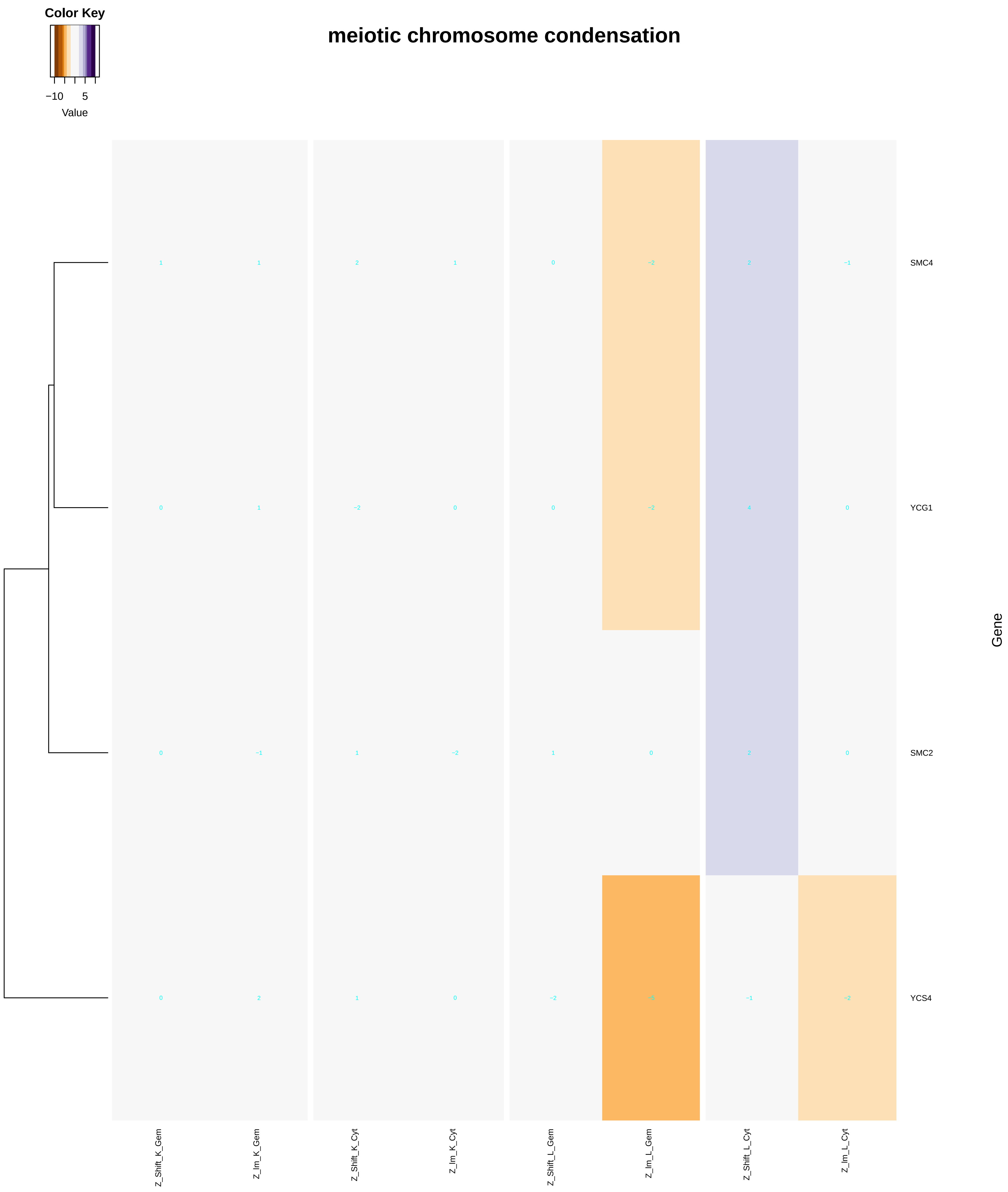

meiotic chromosome condensation
Color Key
−10
5
Value
SMC4
YCG1
SMC2
YCS4
Gene
Z_Shift_K_Gem
Z_Im_K_Gem
Z_Shift_K_Cyt
Z_Im_K_Cyt
Z_Shift_L_Gem
Z_Im_L_Gem
Z_Shift_L_Cyt
Z_Im_L_Cyt
